# Risk Factors for Generalized Anxiety in Older African Americans During the COVID-19 Pandemic

**DOI:** 10.1093/geroni/igaa057.3496

**Published:** 2020-12-16

**Authors:** Dwana Bass, Sophie Hanna, Sarah Shair, Loraine DiCerbo, Bruno Giordani, Voyko Kavcic

**Affiliations:** 1 Wayne State University, Sterling Heights, Michigan, United States; 2 Wayne State University, Detroit, Michigan, United States; 3 University of Michigan, Detroit, Michigan, United States; 4 Wayne State University, Clinton Township, Michigan, United States; 5 University of Michigan, Ann Arbor, Michigan, United States

## Abstract

The COVID-19 pandemic has created worldwide uncertainty and heightened fear and worry, elevating the potential for anxiety. We examined environmental, sociodemographic, and behavioral risk factors predicting generalized anxiety among older African Americans in a large metropolitan area during the COVID-19 outbreak. Sixty African American participants (92% female) age 65 and older were recruited from the Wayne State University Institute of Gerontology Healthier Black Elders Center. In addition to initial demographic questions, the scale (5-point Likert scale from “Not At All” to “A Great Deal”) assessed a range of everyday concerns, such as time spent indoors; family relationships; economic problems; restricted freedom of movement; and access to food, cleaning supplies, medical care, and personal protective equipment during the COVID-19 pandemic. Participants also completed a brief generalized anxiety screener, GAD-7. Of the 60 participants, 5 reported they were COVID-19 infected and their responses were not used for the analyses. Older Black Americans reported that during the pandemic, they were most affected by: Illness in Family (53%), Death in Family (35%) and Isolation (75%). Significant risk factors for anxiety were: Missing Close Ones, Annoyance, Sense of Safety, Media Coverage, and Time Indoors (explaining 58% of variance). The current study highlights everyday risk factors for anxiety in the context of the coronavirus pandemic among city-dwelling African Americans. Identified factors are common concerns that may be ameliorated with reasonable interventions. More research is needed in order to fully understand the scope and correlates of anxiety during these challenging times.

